# Impact of a comprehensive rehabilitation palliative care program on the quality of life of patients with terminal cancer and their informal caregivers: a quasi-experimental study

**DOI:** 10.1186/s12912-024-02028-2

**Published:** 2024-05-29

**Authors:** Ateya Megahed Ibrahim, Nadia Mohamed Ibrahim Wahba, Donia Elsaid Fathi Zaghamir, Nahed Ahmed Mersal, Fathia Ahmed Mersal, Rasmia Abd El-Sattar Ali, Fatma Abdou Eltaib, Heba Ali Hamed Mohamed

**Affiliations:** 1 College of Nursing, Prince Sattam Bin Abdulaziz University, Al-Kharj, 11942 Saudi Arabia; 2 Family and Community Health Nursing Department, Faculty of nursing, Port Said University, Port Said, 42526 Egypt; 3 Psychiatric Nursing and Mental Health Department, Faculty of Nursing, Port Said University, Port Said, 42526 Egypt; 4 Pediatric Nursing Department, Faculty of Nursing, Port Said University, Port Said, 42526 Egypt; 5 Medical Surgical Nursing, Faculty of Nursing, King Abdulaziz University, Jeddah, Saudi Arabia; 6 Community Health Nursing Department, College of Nursing, Northern Border University, Arar, Saudi Arabia; 7 Medical Surgical Nursing Department, Faculty of Nursing, Ain Shams University, Cairo, Egypt; 8 Community Health Nursing Department, Faculty of Nursing, Mansoura University, Mansoura city, Dakahlia Egypt

**Keywords:** Cancer patients, Informal caregivers, Palliative care program, Rehabilitation

## Abstract

**Background:**

Palliative care schemes, which include pain management, symptom control, psychosocial support and rehabilitation, aim to boost patients’ quality of life, ease the burden and anxiety of informal caregivers, and ultimately provide a comprehensive approach to enhance well-being during this challenging and sensitive period. This study aims to evaluate the impact of a comprehensive rehabilitation palliative care program on the quality of life of patients with terminal cancer and their informal caregivers.

**Methods:**

This quasi-experimental study, conducted from August 2023 to January 2024 at outpatient clinics affiliated with the Oncology Center at Mansoura University, Egypt, focused on cancer patients and their caregivers in the palliative care department. Employing pre- and post-test phases, data were gathered using a questionnaire, EORTC QLQ C30, Hospital Anxiety and Depression Scale, Short Form Health Survey, Caregiver Burden Inventory, and Beck Anxiety Inventory. The investigation evaluated a 16-week rehabilitation program comprising exercise, psychoeducation, individual counselling, and spiritual support. Exercises, led by a physiotherapist, targeted fatigue and stress through tailored aerobic and resistance training. Psychoeducation sessions aimed to bolster coping abilities, covering fatigue management and nutrition. Trained counsellors addressed spiritual and existential concerns. Personal advisory sessions were available for individual support. Caregivers received education on rehabilitation and palliative care protocols, ensuring comprehensive patient care.

**Results:**

The mean age for cancer patients was 65.79 ± 13.85. In contrast, the mean age for primary carers was 42.05 ± 11.15. The QOL for cancer patients during the pre-test phase was 77.8 ± 7.16 and rose to 87.34 ± 14.56 during the post-test phase. Additionally, the total anxiety level of patients before the rehabilitation palliative care program was conducted was 15.45 ± 3.05 compared to 6.12 ± 3.21 after the post test phase. Furthermore, the total depression levels of the patients during the pre-test phase were 20.89 ± 9.21. However, after implementing the rehabilitation palliative care program, it decreased to 15.5 ± 6.86. In regards to the total quality of life of informal caregivers, it was measured at 67.28 ± 32.09 before conducting the program. Nevertheless, it increased to 25.95 ± 40.29 after conducting it. Additionally, the total Caregiver Burden Inventory before implementing the program was 37.45 ± 25.7, and it decreased to 29.36 ± 16.4 after conducting it. Additionally, the total score on the Beck Anxiety Inventory decreased from 45.7 ± 4.3 during the initial testing phase to 17.35 ± 23.67.

**Conclusion:**

The program for rehabilitation palliative care successfully achieved its goals by enhancing the overall quality of life for cancer patients and their caregivers. Additionally, it reduced the anxiety and depression levels among the patients, as well as the anxiety and caregiver burden among the caregivers. Continue research into the effectiveness of rehabilitation palliative care programs to identify best practices, improve existing programs, and expand access to these services.

## Background

 Cancer remains a consequential worldwide health issue as its rate continuously rises across the globe [1]. In 2020, the World Health Organization (WHO) approximated that around 19.3 million individuals acquired new cases of cancer. This estimate is projected to be increased to about 28.4 million new cancer cases annually by 2040 [2]. Geographical location, lifestyle factors, and socioeconomic status are factors that significantly affect the incidence of cancer [3]. Developed countries frequently have higher rates of cancer incidence, influenced by factors such as an aging population, exposure to environmental pollutants, and changes to lifestyle habits [4]. Efforts to tackle the increasing prevalence of cancer worldwide include the promotion of healthy living practices, screening programs that enable the early detection of cancer and advances in research and development for cancer treatment [5, 6].

The incidence of cancer in Arab countries has been increasing, presenting a mounting health challenge in the region [7]. Based on 2020 data from the Global Cancer Observatory (GLOBOCAN), the estimated age-standardized incidence rate of all cancers in Arab countries was 133.4 per 100,000 individuals, with variations among different nations [8]. This rise is linked to multiple factors, such as lifestyle changes, environmental exposures, and unequal healthcare access [9]. Specific cancers, including breast, liver, and lung cancer, have considerably contributed to the overall incidence of cancer in these regions [10]. The increase in incidence emphasises the significance of implementing holistic approaches to cancer prevention, early detection and improving access to quality cancer care to address this crucial public health issue in Arab countries [7].

As of the latest update in January 2022, Egypt is facing an increasingly significant cancer burden, with statistics underscoring the magnitude of the issue [11]. According to GLOBOCAN 2020 data, the age-standardized incidence rate for all cancer types in Egypt was 126.9 per 100,000 individuals [8]. Egypt has a notably high prevalence of Hepatitis C virus (HCV) infection, leading to a significant incidence of hepatocellular carcinoma (liver cancer) [12]. Furthermore, breast cancer, lung cancer, and colorectal cancer are major contributors to cancer statistics within the country. Efforts within the field of public health have been focused on increasing awareness, detecting cases early, and improving accessibility to cancer care services in order to alleviate the impact of cancer on the population of Egypt [11].

The impact of a comprehensive rehabilitation palliative care programme on the quality of life of patients with terminal cancer and their informal caregivers is a highly significant topic [13]. When faced with a terminal cancer diagnosis, individuals undertake a challenging and emotionally taxing journey that requires a multifaceted care approach [14]. Rehabilitation Palliative care programmes are devised to fulfil the intricate and ever-changing requirements of these patients, providing a complete framework of assistance that covers their physical, emotional and psychosocial well-being [15].

Patients diagnosed with terminal cancer often grapple with excruciating physical symptoms and the emotional turmoil that accompanies their condition [16]. In this context, comprehensive rehabilitation palliative care programs come to the forefront as a beacon of hope [17]. These programs aim to alleviate the burdens carried by patients by providing effective pain management and symptom control. By doing so, they contribute significantly to enhancing the physical comfort and overall quality of life for patients as they navigate their illness [18].

The effects of such programs extend beyond the physical realm. The emotional and psychological difficulties related to terminal cancer cannot be disregarded [19]. Rehabilitation Palliative care programmes give essential emotional support, providing patients with the tools to manage the emotional obstacles that come with a terminal diagnosis [20]. The ensuing emotional well-being and psychological comfort, ultimately, add to an increased overall quality of life for patients [21].

Additionally, clear and objective communication alongside collaborative decision-making play crucial roles in rehabilitation palliative care [22]. It is crucial to encourage patients to actively participate in making decisions about their care to promote a sense of control, dignity, and enhance their quality of life [23]. The involvement of informal caregivers in the decision-making process also has significant benefits, empowering them and relieving any emotional distress they may feel [24].

One of the key features of rehabilitation palliative care is the development of bespoke care plans. These plans are carefully created with the individual needs and preferences of patients in mind, ensuring that their care is in line with their values and objectives [25]. This personalised approach results in care that not only enriches the patients’ quality of life, but also provides reassurance to caregivers, as they can be confident that their loved ones are receiving optimal care [26].

Recognising the spiritual and existential aspects of care is an additional essential element of palliative and rehabilitation care [27]. These programmes assist patients and caregivers to discover meaning and purpose when faced with a terminal illness, resulting in a greater sense of peace and well-being [28]. This comprehensive approach goes beyond the physical and emotional domains to address the deeper spiritual needs of those dealing with terminal cancer [29].

Comprehensive rehabilitation palliative care programmes also acknowledge the essential role of informal caregivers, who often bear the emotional and physical burden of providing care [30]. These programmes provide these carers with resources, education, and respite, thus mitigating the risk of burnout and promoting their well-being. This, in turn, results in a notable enhancement of carers’ overall quality of life [31].

Furthermore, rehabilitation palliative care programmes frequently provide assistance to caregivers in the post-bereavement phase following a patient’s demise [15]. This assistance assists caregivers in managing the grieving process, coping with loss, and finding comfort. Such programmes guarantee that caregivers can maintain a better quality of life even after their loved one has departed, by providing ongoing support following their passing [30].

Ultimately, exhaustive rehabilitation palliative care schemes provide an all-encompassing and sympathetic strategy to end-of-life care [32]. They acknowledge and tackle not simply the patient’s physical and emotional elements but also their social, spiritual, and existential facets [13]. By tailoring care to the individual needs of patients and acknowledging the significant role of informal caregivers, these programmes greatly enhance the quality of life of patients with terminal cancer and their caregivers [33]. This comprehensive approach ensures that individuals facing the challenging journey of terminal cancer receive the necessary support and resources to maintain a superior quality of life [25].

Community health nurses are essential to the success of a comprehensive program for rehabilitation palliative care of patients with terminal cancer and their informal caregivers. Their importance cannot be overstated [34]. They act as intermediaries between healthcare institutions and the community, giving vital assessments, education, emotional support, and symptom management. Community health nurses enhance the physical and emotional well-being of patients and provide support, guidance, and resources to reduce caregiver burden and maintain their well-being [35].

They achieve this by promoting shared decision-making, advocating for patients’ and caregivers’ needs, and ensuring effective communication within the healthcare team. These nurses have a crucial part to play in enhancing the quality of life for individuals dealing with the difficulties of terminal cancer and their devoted caretakers [34]. They accomplish this by applying objective measures to ensure that their patients are comfortable, and by providing necessary support to their caregivers. This not only improves the healthcare outcomes but also contributes to an overall enhanced living experience for individuals facing such challenges [35]. Thus, the present study aimed to assess the effect of an inclusive rehabilitation palliative care initiative on the well-being of patients diagnosed with advanced-stage cancer and their non-professional caregivers.

### Aim of the study

To evaluate the impact of a comprehensive rehabilitation palliative care program on the quality of life of patients with terminal cancer and their informal caregivers.

**Research objectives**:

1. Assess the effectiveness of a 16-week rehabilitation program in improving the physical well-being of cancer patients undergoing treatment at the Damietta Cancer Institute’s palliative care department.
2. Evaluate the impact of psychoeducation sessions on enhancing coping abilities and reducing anxiety and depression among cancer patients and their primary caregivers.
3. Investigate the role of spiritual and existential support in enhancing the overall quality of life and emotional well-being of cancer patients and their caregivers during palliative care.

**Research questions**:

1. How does the comprehensive rehabilitation palliative care program at the Oncology Centre, Mansoura University impact the overall well-being and quality of life for cancer patients?
2. In what ways do primary caregivers perceive changes in their roles and experiences as a result of participating in the rehabilitation program alongside cancer patients in the palliative care department?
3. To what extent does the rehabilitation palliative care initiative foster improved communication, satisfaction, and holistic care experiences for both patients and caregivers at the Oncology Centre, Mansoura University?

## **Methods**

### Research design

This study adopts a quasi-experimental design, incorporating both pre- and post-test phases. Quasi-experimental studies are particularly useful for assessing the impact of educational interventions as they allow for the observation of changes within the same group over time. This design is advantageous for eliminating potential confounding effects of socio-demographic factors on the study’s outcomes, thereby providing a robust framework for evaluating intervention effectiveness.

### Study setting

This study took place within the outpatient clinics affiliated with the Oncology Center at Mansoura University, situated in the Delta region of Egypt. The center serves as a vital healthcare hub for individuals grappling with cancer diseases, offering comprehensive treatment modalities and advanced preventive services to patients across the Delta and Channel governorates. Its overarching goal is to provide integrated care for various oncological disciplines, encompassing digestive, liver, blood, bone, and marrow transplant cases. Additionally, the center is equipped with state-of-the-art radiology, analysis, and pathology facilities, further enhancing its capacity to address the multifaceted needs of cancer patients. In this setting, the study aimed to explore and address pertinent issues related to cancer care and treatment efficacy, thereby contributing to the advancement of oncological practices and patient outcomes within the region.

### Subjects

The participants in this study comprised cancer patients and their primary caregivers who met specific inclusion criteria. Cancer patients were required to be 18 years of age or older and have a history of stage III or IV cancer. Additionally, they should have no communication problems or psychiatric diagnoses and be willing to participate in the study. Exclusion criteria for patients included physical risk due to cancer or severe comorbidities, prohibitive psychopathology, serious cognitive disturbances, or medication side effects. Caregivers eligible for inclusion were aged between 18 and 60 years, literate, providing primary care to third- or fourth-stage cancer patients, and had no communication issues or psychiatric diagnoses. Like patients, caregivers also needed to volunteer for participation in the study. These stringent criteria ensured that the study population was representative of individuals who could actively engage with the intervention and provide valuable insights into its effectiveness.

### Sample size and sampling technique

The study’s sample size was determined using G*Power 3 software, employing a combination of statistical parameters to ensure robustness. With a confidence level of 95% (1-α), a test power of 95.4% (1-β), and an effect size (d) of 0.92, the sample size calculation aimed to achieve adequate statistical power for detecting meaningful differences. The sampling technique utilized was purposive sampling, whereby participants were selected based on predetermined criteria to ensure representation of the target population. This approach facilitated the recruitment of cancer patients and primary caregivers who met specific eligibility criteria, such as age, cancer stage, absence of communication problems or psychiatric diagnoses, and willingness to participate. By employing purposive sampling, the study aimed to recruit a sample that could provide valuable insights into the effectiveness of the intervention while ensuring the study’s feasibility and practicality within the research context.

### Data collection measures

Data collection measures spanned from August 2023 to January 2024, during which five tools were employed:

### Questionnaire forum

Two questionnaire forms were distributed, one for patients and the other for caregivers. The researchers developed the questionnaire forms, with reference to literature to ensure objectivity. The questionnaire consisted of socio-demographic questions on patients and caregivers including age, sex, marital status, and education. Furthermore, it contained inquiries about disease characteristics, and duration of care.

### EORTC QLQC30

The EORTC QLQ-C30, validated and recognized for its reliability in Arabic by Jassim and Al-Ansari [36], constitutes a pivotal self-report instrument meticulously crafted for cancer patients. Comprising a total of 30 meticulously designed items, this questionnaire delves into multifaceted dimensions crucial for the meticulous evaluation of quality of life (QOL). These dimensions encompass a global quality of life (QOL) scale, in addition to five distinct functional scales (physical, role, cognitive, emotional, and social), as well as an exhaustive array of nine symptom scales (fatigue, pain, nausea, constipation, diarrhea, insomnia, dyspnea, financial difficulties, and loss of appetite). It is noteworthy that respondents are prompted to assess the severity or frequency of their experiences through a four-point Likert scale across the initial 28 items, while the final two items (29 and 30) are graded on a scale spanning from 1 to 7 points. An elevation in scores within the functional domain and the terminal two items typically denotes an ameliorated quality of life, whereas a heightened score in symptom management correlates with a perceived decline in QOL. Widely regarded as a reliable and valid instrument, the EORTC QLQ-C30 represents a valuable resource for the critical evaluation of clinical and psychosocial interventions in the context of cancer care [37–40]. Of note, the questionnaire demonstrated commendable internal consistency within the present study, as evidenced by a Cronbach’s alpha coefficient of 0.83.

### Hospital anxiety and depression scale (HADS)

The Hospital Anxiety and Depression Scale (HADS) has been meticulously validated and proven to be reliable in Arabic by Al Aseri et al. [41]. This comprehensive 14-item questionnaire is purposefully crafted to discern symptoms of anxiety and depression among patients. Initially devised as a convenient and dependable screening tool for healthcare practitioners, the HADS has undergone thorough validation processes, affirming its efficacy for screening purposes. Demonstrating effectiveness across diverse settings, including somatic and psychiatric contexts, primary care environments, and broader population studies, the HADS has exhibited its utility in assessing the severity and frequency of anxiety disorders and depression cases. Consequently, healthcare professionals are empowered to confidently employ the Arabic version of HADS as a valuable instrument for pinpointing and evaluating symptoms of anxiety and depression in both clinical practice and research endeavors. Moreover, the HADS, widely esteemed as a reliable and valid instrument, stands as an invaluable resource for critically evaluating clinical and psychosocial interventions in the realm of cancer care [40, 42]. Notably, within the confines of the present study, this questionnaire demonstrated impressive internal consistency, as reflected by a Cronbach’s alpha coefficient of 0.89.

### Short form health survey (SF-36)

The Short Form Health Survey (SF-36) scale, devised by Ware and Sherbourne [43], has been rigorously validated and proven reliable in Arabic by Guermazi et al. [44], comprising 36 items, this scale encompasses eight distinct sub-scales, each designed to assess various facets of health and well-being, including physical functioning, role physical, energy/fatigue, bodily pain, general health perceptions, social functioning, role emotional, and mental health. Additionally, the SF-36 yields two main dimensions: physical and mental health. The quality of life is positively correlated with elevated scores on both the overall scale and its individual sub-scales. Notably, the internal consistency of each sub-scale is high, with Cronbach’s alpha coefficients ranging from 0.83 to 0.86. As such, healthcare professionals can confidently utilize the Arabic version of the SF-36 as a robust tool for evaluating health-related quality of life across diverse populations and settings.

### Caregiver burden inventory (CBI)

The Caregiver Burden Inventory (CBI) has been rigorously validated and shown to be reliable in Arabic by Mohamed Mahmoud, Helmy Osman, Mohamed El-Sayed Gaafar, & Ibrahim Hassan Gomaa[45], Developed by Novak and Guest [46], this scale comprises 24 items and is designed to assess the impact of caregiving on caregivers across five subscales: time-addiction burden, developmental burden, physical burden, social burden, and emotional burden. Scores on the inventory range from 0 to 100, with higher scores indicating a greater burden on caregivers and lower scores indicating less burden. In the present study, the validity and reliability of the Arabic version of the CBI were both reported to be 0.95, indicating robust psychometric properties. This underscores the utility of the Arabic CBI as a valid and reliable instrument for evaluating caregiver burden in Arabic-speaking populations.

### Beck anxiety inventory (BAI)

The Arabic version of the Beck Anxiety Inventory (BAI) has been validated and deemed reliable by Abdel-Khalek [47]. Originally developed by Beck, Epstein, Brown, and Steer [48], the BAI is designed to assess the frequency of anxiety symptoms among caregivers. Consisting of 21 items, each scored between 0 and 3, higher scores on the BAI indicate elevated levels of anxiety in individuals. In the present study, the validity and reliability of the Arabic version of the scale were reported to range between 0.88 and 0.89, affirming its robust psychometric properties and suitability for evaluating anxiety symptoms among Arabic-speaking caregivers.

### Rehabilitation palliative care intervention program

Cancer patients and their caregivers participated in a 16-week rehabilitation program, which included exercise, psychoeducation, individual counselling, and, importantly, components addressing spiritual and existential concerns[49]. The program commenced with a qualified physiotherapist conducting 60-minute exercise sessions for four times. These sessions included aerobic and resistance training, such as walking, cycling, and rowing, tailored to each participant’s baseline physical capacity. Alongside physical training, sessions also focused on managing symptoms like fatigue and stress, progressively adapting to individual needs[50].

The second component, psychoeducation, was delivered four times over the program’s duration, each session lasting 40 min. These sessions aimed to boost patients’ self-confidence and autonomy, offering tailored support for coping with the cancer diagnosis and its side effects. Topics covered included managing fatigue, physical limitations, anxiety, stress, job reintegration, and nutrition.

In addition to these components, we integrated four sessions specifically designed to address the spiritual and existential challenges faced by patients and caregivers. Recognizing the profound impact of a cancer diagnosis on patients’ spiritual well-being, we included guided discussions facilitated by trained counselors skilled in spiritual care. These discussions aimed to provide comfort, explore personal values and beliefs, and offer support through spiritual reflection and connection, which are pivotal in enhancing the quality of life during palliative care.

The program, personal advisory, offered individualized support and was available at the start of each physical exercise session and at the conclusion of the program. These 10-minute sessions allowed for addressing inquiries, providing guidance, and ensuring each patient’s and caregiver’s unique spiritual and existential needs were met.

For caregivers, the educational planning included specific sections on rehabilitation and palliative care protocols, with comprehensive guidance on general body care, nutrition, physical exercise, psychological support, medication, and symptom control[51–53]. The manual, evaluated for clarity and precision by an oncologist and four nurses, also incorporated discussions on spiritual support, helping caregivers address both their own and the patients’ spiritual needs. Each section was tailored for discussion within the palliative care protocol, recognizing the diversity of caregivers’ needs and ensuring a holistic approach to care (Table 1).

**Table 1:** Session Content for rehabilitation palliative care program

Session	Focus	Description
1	Introduction	Introduction to the program, understanding patient and caregiver needs, initial assessments.
2	Physical Training	Begin physical training with basic exercises focusing on cardio and resistance; address physical symptoms like fatigue.
3	Psychoeducation	Discuss managing fatigue, physical limitations; introduce stress and anxiety management techniques.
4	Spiritual Support	Guided discussion on spiritual wellbeing, exploring personal values and beliefs, offering spiritual comfort.
5	Physical Training	Increase intensity of exercises; focus on symptom management and individual adaptation of exercises.
6	Psychoeducation	Cover topics on job reintegration, nutrition, and long-term symptom management strategies.
7	Spiritual Support	Continue spiritual discussions, deepen reflection, and support spiritual connection.
8	Review and Assess	Evaluate progress, adjust physical and psychoeducation plans, reassess spiritual and existential needs.
9	Physical Training	Further adapt and tailor physical training to patient feedback and progress.
10	Psychoeducation	Focus on advanced coping strategies, dealing with chronic stress, and preparing for end-of-life care.
11	Spiritual Support	Guided discussion on spiritual wellbeing, exploring personal values and beliefs, offering spiritual comfort.
12	Physical Training	Increase intensity of exercises; focus on symptom management and individual adaptation of exercises.
13	Psychoeducation	Cover topics on job reintegration, nutrition, and long-term symptom management strategies.
14	Spiritual Support	Guided discussion on spiritual wellbeing, exploring personal values and beliefs, offering spiritual comfort.
15	Review and Assess	Evaluate progress, adjust physical and psychoeducation plans, reassess spiritual and existential needs.
16	Closure	Review overall progress, provide guidelines for continued care, final assessments.

### Delivery of the intervention: recruitment, training, and roles of interventionists

#### Recruitment of interventionists

The intervention program involved a diverse team of qualified professionals, each selected based on their expertise in relevant fields such as physiotherapy, psychoeducation, counselling, and spiritual care. The recruitment process for these interventionists was rigorous and aimed at ensuring that only individuals with significant experience and appropriate credentials were chosen. Specific details of the recruitment process included:

1. **Physiotherapists**: Candidates were required to have a background in oncology rehabilitation, demonstrating proficiency in designing and implementing exercise programs tailored for cancer patients.
2. **Counsellors**: Professionals with qualifications in psychology or counselling, particularly with experience in dealing with terminal illnesses and palliative care, were selected. Special emphasis was placed on their ability to address spiritual and existential concerns.
3. **Educators for Psychoeducation Sessions**: These were individuals with expertise in patient education, ideally with experience in oncology or chronic disease management, to provide relevant and impactful sessions.

#### Training of interventionists

Once recruited, the interventionists underwent comprehensive training to ensure consistency and effectiveness in delivering the program. The training process included:

1. **Standardized Protocols**: All interventionists were trained on standardized protocols to ensure uniformity in the intervention delivery. This included detailed guidelines on the exercise routines, psychoeducation content, counselling methods, and spiritual care practices.
2. **Workshops and Seminars**: Regular workshops and seminars were conducted to update the interventionists on the latest evidence-based practices in palliative care and rehabilitation. These sessions also provided a platform for interventionists to share experiences and strategies.
3. **Role-Playing and Simulation**: To prepare for real-world interactions, interventionists participated in role-playing and simulation exercises. This training was crucial for honing their skills in handling diverse patient and caregiver scenarios effectively.
4. **Supervision and Feedback**: Throughout the training period, interventionists received supervision and feedback from senior practitioners. This iterative process ensured continuous improvement and alignment with the program’s objectives.

#### Delivery of the intervention

The comprehensive rehabilitation palliative care program was structured into several key components, each delivered by specific professionals:

1. **Physical exercise sessions:**
  Lead by physiotherapists: These sessions were conducted by qualified physiotherapists who tailored aerobic and resistance training exercises to each participant’s baseline physical capacity. The exercises aimed at improving physical strength and managing symptoms such as fatigue and stress.
  Frequency and Duration: The exercise sessions were held four times over the 16-week program, each lasting 60 min.
2. **Psychoeducation Sessions:**
  Delivered by educators: These 40-minute sessions were facilitated by educators experienced in patient education, focusing on building self-confidence and autonomy in managing the cancer diagnosis and its side effects.
  Content: Topics covered included coping strategies for fatigue, anxiety, stress, physical limitations, job reintegration, and nutrition.
3. **Spiritual and existential care:**
  Facilitated by trained counsellors: Recognizing the profound impact of a cancer diagnosis on spiritual well-being, trained counsellors conducted sessions addressing spiritual and existential challenges.
  Guided discussions: These discussions provided comfort, explored personal values and beliefs, and offered support through spiritual reflection and connection.
4. **Personal advisory sessions:**
  Individualized support:At the start of each physical exercise session and at the program’s conclusion, 10-minute personal advisory sessions were held to address specific inquiries and provide tailored guidance.
  Meeting unique needs: These sessions ensured that both patients’ and caregivers’ unique spiritual and existential needs were met.
5. **Caregiver Education:**
  Comprehensive guidance:Educational sessions for caregivers included rehabilitation and palliative care protocols, focusing on body care, nutrition, physical exercise, psychological support, medication, and symptom control.
  Manual for caregivers: A manual, reviewed for clarity by an oncologist and four nurses, was provided. It included detailed discussions on spiritual support to help caregivers address their own and the patients’ spiritual needs.

#### Evaluation and feedback

Throughout the intervention, regular assessments and feedback mechanisms were in place to monitor the effectiveness of the program and make necessary adjustments. This approach ensured that the intervention remained patient-centred and responsive to the evolving needs of both patients and caregivers. By rigorously recruiting and training skilled professionals and implementing a structured, multidisciplinary approach, the rehabilitation palliative care program was able to significantly improve the quality of life for terminal cancer patients and their informal caregivers.

### Procedure

In the comprehensive evaluation conducted at outpatient clinics affiliated with the Oncology Center at Mansoura University, Egypt, an elaborate procedure was devised to meticulously gauge the effectiveness of a 16-week rehabilitation program tailored for cancer patients and their primary caregivers. The process commenced with the collection of baseline data through pre-intervention questionnaires, systematically administered to participants before the implementation of the rehabilitation initiative. These questionnaires, spanning instruments such as the EORTC QLQ-C30, Hospital Anxiety and Depression Scale (HADS), Short Form Health Survey (SF-36), Caregiver Burden Inventory (CBI), and Beck Anxiety Inventory (BAI), served as the foundational tools for assessing a wide array of parameters pertaining to participants’ quality of life, psychological well-being, and caregiver burden.

Following the meticulous acquisition of baseline data, the subsequent phase of the study transitioned seamlessly into the post-intervention period, marked by the immediate initiation of data collection upon the conclusion of the 16-week rehabilitation program. This post-intervention data collection mirrored the structure and content of the pre-intervention assessments, employing the same set of questionnaires to capture participants’ evolving states and experiences following the completion of the intervention. This meticulous alignment in data collection time points allowed for a robust comparison between pre and post-intervention measures, enabling researchers to discern and quantify the precise impact of the rehabilitation program on participants’ outcomes.

By orchestrating this intricately choreographed procedure, the study sought to unravel the nuanced dynamics and efficacy of the rehabilitation intervention in ameliorating the physical, emotional, and psychological challenges faced by cancer patients and their caregivers. Through the meticulous collection and analysis of data at distinct time points, the study aimed to furnish valuable insights into the tangible benefits and transformative potential of tailored rehabilitation initiatives in enhancing the holistic well-being of individuals navigating the complex terrain of cancer care.

### Ethical considerations

This study complies with the Declaration of Helsinki guidelines on an ethics committee opinion for approval. An approval was obtained from the Research Ethics Committee of the Faculty of Nursing at Mansoura University under code number (0584). Participants were provided with an explanation of the study’s purpose prior to being asked to provide an informed written consent to participate and written informed consent was obtained from all the participants and for illiterate participants the informed consent was obtained from their legal guardian or legally authorized representatives. A short summary was also provided to reassure the participants that any data collected would be kept confidential and used solely for research purposes. Participants were duly informed of their right to participate or withdraw from the study at any time. For identification purposes, code numbers were used instead of participant names. This safeguarded participants’ anonymity in public reports.

### Data analysis

Data were analyzed using IBM SPSS 24 software. Descriptive data were presented as numbers, percentages, means and standard deviations. The Wilcoxon signed-rank test was used to compare baseline and end-of-program values, as an alternative to the paired Student’s t-test when the population cannot be assumed to be normally distributed. The mean was reported as the standard deviation for normally distributed quantitative data and the median (min-max) for non-normally distributed data. Based on the results, the confidence interval was set at 95% and the significance level was set at *p* < 0.05.

## Results

Table 1 presents the distribution of patients and their family caregivers based on demographic data and disease characteristics of the patients. The data illustrates a diverse cohort, with patients spanning various ages and cancer types, reflective of the complex landscape of oncological care. The mean age of patients was 65.79 years, with a standard deviation of 13.85, while caregivers had a mean age of 42.05 years, with a standard deviation of 11.15. In terms of gender distribution, the majority of patients were male (54.5%), whereas females constituted 45.5%. Conversely, among caregivers, females represented a larger proportion (59.1%) compared to males(49%). Regarding marital status, the majority of patients were married (71.6%), whereas a significant proportion of caregivers were single (42%). Education status varied among participants, with a notable portion of patients having low educational attainment (44.3%) and caregivers distributed across low (17%), moderate (54.5%), and high (28.5%) education levels. The distribution of cancer types depicted diversity, with breast, colon, liver, and lymphoma/leukaemia representing notable proportions.

The duration since diagnosis varied, with a significant portion diagnosed within 7 to 12 months (51.1%). Most patients were at Stage 3 of cancer (53.4%), with various treatment modalities utilized, including chemotherapy (56.8%) and radiotherapy (22.7%). The duration of caregiving revealed a split between those providing care for less than 12 months (40.9%) and those providing care for 12 months or more (59.1%).

Table 2 presents a comparison of mean scores on the European Organization for Research and Treatment of Cancer Quality of Life Questionnaire (EORTC QLQ-C30) and the Hospital Anxiety and Depression Scale (HADS) among cancer patients before and after receiving palliative care and rehabilitation education. The table includes the post-test and pre-test mean scores, standard deviations (SD), and statistical analysis results using the Wilcoxon signed-rank test. For the EORTC QLQ-C30 scale, significant improvements were observed in various domains post-intervention compared to pre-intervention scores. Notably, there were significant improvements in quality of life (post-test mean = 62.73 vs. pre-test mean = 44.65, *p* < 0.05), physical functioning (post-test mean = 91.36 vs. pre-test mean = 76.9, *p* < 0.05), role functioning (post-test mean = 66.77 vs. pre-test mean = 46.92, *p* < 0.05), emotional functioning (post-test mean = 79.62 vs. pre-test mean = 68.23, *p* < 0.05), cognitive functioning (post-test mean = 63.24 vs. pre-test mean = 54.68, *p* < 0.05), and social functioning (post-test mean = 70.23 vs. pre-test mean = 51.71, *p* < 0.05). Significant improvements were also observed in symptom control domains, including fatigue, nausea, pain, dyspnoea, insomnia, appetite loss, constipation, diarrhoea, and financial difficulties (all *p* < 0.05).Similarly, for the HADS scale, significant reductions in anxiety and depression symptoms were observed post-intervention compared to pre-intervention scores. The mean scores for anxiety decreased from 15.21 pre-intervention to 5.61 post-intervention (*p* < 0.05), while the mean scores for depression decreased from 18.39 pre-intervention to 3.18 post-intervention (*p* < 0.05).

**Table 2:** Distribution of patients and their family caregivers according to the demographic data and disease characteristics of patients

Characteristics	Patient *n* (%)	Caregiver *n* (%)
**Age**	65.79 ± 13.85	42.05 ± 11.15
**Sex**		
Male	**48 (54.5)**	**36 (40.9)**
Female	**40 (45.5)**	**52 (59.1)**
**Marital status**		
Single	**25 (28.4)**	**37** (42)
Married	**63 (71.6)**	**51** (58)
**Education status**		
Cannot read and write	**39 (44.3)**	**-**
Low education	**20 (22.7)**	**15** (17)
Moderate education	**17 (19.3)**	**48(54.5)**
High education	**12 (13.6)**	**25 (28.5)**
**Type of cancer**		
Breast	**23 (26.1)**	**-**
Colon	**17 (19.3)**	**-**
Liver	**29** (33)	**-**
Lymphoma and leukemia	**5 (5.7)**	**-**
Lungs	**4 (4.5)**	**-**
Hematological	**4 (4.5)**	**-**
Testicular	**2 (2.3)**	
Ovarian	**3 (3.4)**	
Brain	**1 (1.2)**	
**Time since diagnoses (in months)**		
0 to 6	**28 (31.8)**	**-**
7 to 12	**45 (51.1)**	**-**
> 12	**15 (17.1)**	**-**
**Stage of cancer**		
Stage 3	**47 (53.4)**	**-**
Stage 4	**41 (46.6)**	**-**
**Type of treatment**		
Hormonal therapy	**13 (14.8)**	**-**
Surgery	**5 (5.7)**	
Chemotherapy	**50 (56.8)**	
Radiotherapy	**20 (22.7)**	
**Duration for providing care**		
< 12 months	**-**	**36 (40.9)**
≥ 12 months	**-**	**52 (59.1)**

Tables 3 and 4 depicts a comparison of mean scores on the Short-Form Health Survey (SF-36), Caregiver Burden Inventory (CBI), and Beck Anxiety Inventory (BAI) among primary caregivers before and after participating in rehabilitation palliative care and training. The table includes post-test and pre-test mean scores, standard deviations (SD), and the results of statistical analysis using the Wilcoxon signed-rank test. For the SF-36 scale, significant improvements were observed in various domains post-intervention compared to pre-intervention scores. Notably, there were significant improvements in physical functioning (post-test mean = 66.57 vs. pre-test mean = 42.15, *p* < 0.05), role physical (post-test mean = 57.38 vs. pre-test mean = 33.16, *p* < 0.05), role functioning (post-test mean = 63.18 vs. pre-test mean = 37.58, *p* < 0.05), bodily pain (post-test mean = 49.89 vs. pre-test mean = 27.59, *p* < 0.05), general health (post-test mean = 54.69 vs. pre-test mean = 55.59, *p* < 0.05), vitality (post-test mean = 45.78 vs. pre-test mean = 49.58, *p* < 0.05), social functioning (post-test mean = 73.98 vs. pre-test mean = 60.33, *p* < 0.05), role emotional (post-test mean = 67.25 vs. pre-test mean = 46.29, *p* < 0.05), mental health (post-test mean = 65.62 vs. pre-test mean = 45.92, *p* < 0.05), and total SF-36 scores (post-test mean = 25.95 vs. pre-test mean = 67.28, *p* < 0.05).

**Table 3:** Comparison of EORTC QLQ-C30 and HADS mean scores among cancer patients before and after palliative care and rehabilitation education

EORTC QLQ-C30 Scale	Pre-test		Post-test		Z	*P*
	X	SD	X	SD		
Quality of life	44.65	11.16	62.73	13.92	–4.25	0.000
Physical functioning	76.9	35.20	91.36	18.59	–5.892	0.000
Role functioning	46.92	48.25	66.77	25.24	–3.322	0.000
Emotional functioning	68.23	19.32	79.62	28.37	–1.092	0.000
Cognitive functioning	54.68	23.08	63.24	22.19	–2.952	0.000
Social functioning	51.71	42.25	70.23	19.35	–4.73	0.000
Fatigue	45.35	23.17	32.81	29.81	–1.556	0.000
Nausea	9.28	8.08	7.51	11.49	–2.91	0.000
Pain	41.43	26.17	20.53	17.83	–3.368	0.000
Dyspnea	26.7	19.54	17.18	33.27	–4.652	0.000
Insomnia	72.09	55.43	42.43	32.65	–3.614	0.000
Appetite loss	35.28	22.7	17.45	28.14	–3.415	0.000
Constipation	16.78	17.55	5.61	15.21	–1.706	0.000
Diarrhea	17.32	25.28	3.18	18.39	–2.615	0.000
Financial difficulties	35.17	22.10	4.15	15.56	–3.75	0.000
**EORTC OLQ-C30 total scores**	77.8	7.16	87.34	14.56	-17.87	0.000
**HADS**						
Anxiety	15.45	3.05	6.12	3.21	–1.452	0.000
Depression	20.89	9.21	15.5	6.86	–3.735	0.000

EORTC QLQ-C30—European organization for research and treatment of cancer quality, *HADS* Hospital anxiety and depression scale

Z— Wilcoxon signed-rank test. *p*<0.05 indicate significance

**Table 4:** Comparison of Short-Form Health Survey (SF-36), caregiver burden inventory (CBI), and beck anxiety inventory (BAI) mean scores of primary caregivers pre- and post-rehabilitation palliative care and training

SF-36	Pre-test		Post-test		Z	*P*
	X	SD	X	SD		
Physical functioning	42.15	18.68	66.57	19.54	–4.518	0.000
Role physical	33.16	37.25	57.38	15.73	–8.165	0.000
Role functioning	37.58	21.21	63.18	44.36	–5.016	0.000
Bodily pain	27.59	51.47	49.89	51.19	–2.562	0.000
General health	55.59	17.41	54.69	30.62	–2.106	0.000
Vitality	49.58	16.52	45.78	19.18	–4.301	0.000
Social functioning	60.33	20.26	73.98	16.48	–1.281	0.000
Role emotional	46.29	15.34	67.25	22.43	–3.365	0.000
Mental health	45.92	18.63	65.62	13.61	–2.351	0.000
**Total SF-36**	67.28	32.09	25.95	40.29	-9.348	0.000
***CBI***						
Time-addiction burden	43.56	82.2	31.78	15.5	-1.345	0.000
Developmental burden	32.14	12.45	19.34	23.9	-2.456	0.000
Physical burden	44.65	10.34	21.34	12.30	-3.981	0.000
Social burden	27.67	9.32	14.67	10.34	-1.876	0.000
Emotional burden	39.23	6.2	21.8	30.2	-2.45	0.000
** Total CBI score**	37.45	25.7	29.36	16.4	-2.411	0.000
** Total BAI Score**	45.7	4.3	17.35	23.67	-5.321	0.000

*SF-36 *Short-Form Health Survey, *CBI* Caregiver burden inventory, *BAI *Beck anxiety inventory

Z— Wilcoxon signed-rank test. *p*< 0.05 indicate significance

Similarly, for the CBI scale, significant reductions in caregiver burden were observed post-intervention compared to pre-intervention scores. The mean scores for time-addiction burden, developmental burden, physical burden, social burden, emotional burden, and total CBI score all showed significant decreases post-intervention (all *p* < 0.05). Additionally, for the BAI scale, significant reductions in anxiety symptoms were observed post-intervention compared to pre-intervention scores. The mean total BAI score decreased significantly from pre-intervention to post-intervention (*p* < 0.05).

## Discussion

A comprehensive rehabilitation palliative care programme can have a profound and positive impact on the quality of life of patients with terminal cancer and their informal caregivers [17]. This holistic approach not only addresses the physical symptoms and pain management associated with cancer, but also focuses on the emotional, psychological and social aspects of care [54]. By providing a support system that includes pain control, symptom management, psychological counselling, and assistance with activities of daily living, such a programme can significantly improve the overall well-being of the patient, allowing them to maintain a higher level of functioning and independence [55]. It also eases the burden on informal carers by providing guidance, respite care and emotional support, thereby improving their own quality of life and reducing the stress and anxiety that often accompanies caring for a loved one with a terminal illness [13]. Overall, a comprehensive rehabilitation and palliative care programme plays a vital role in improving the quality of life for both patients and their carers during the challenging journey of terminal cancer [17].

The results of the study, which showed a significant improvement in the overall quality of life (QOL) of cancer patients following the implementation of a palliative care rehabilitation programme, highlight the critical role of comprehensive care in addressing the complex needs of people facing terminal cancer. In addition, palliative care, when integrated with rehabilitation, focuses on improving the physical, emotional and social well-being of patients, which naturally contributes to improved QOL. Several studies and clinical trials have also reported significant benefits associated with palliative care programmes. For example, a study by Franciosi, et al. [56] found that early palliative care intervention led to improved quality of life, reduced symptoms and increased survival in patients with advanced lung cancer.

Also, this finding is supported by (Groh, Vyhnalek, Feddersen, Führer, & Borasio) [57], who reported that the involvement of a specialised outpatient palliative care team leads to a significant improvement in the quality of life of patients and caregivers and can reduce the burden of home care for caregivers of seriously ill patients. Furthermore, (Bani Younis, Al-Rawashdeh, Alnjadat) [58] concluded that the results showed a statistically significant effect on the quality of life domains. For the workshop experimental group and for the home visiting experimental group.

Consistently, these findings are in line with existing research demonstrating that palliative care, with its holistic approach to patient care, can effectively alleviate suffering and improve the QOL of individuals facing life-limiting illness [59, 60]. Furthermore, this comprehensive care strategy has been shown to positively impact patients’ QOL by addressing their pain management, symptom control and psychosocial needs [61, 62].

Moreover, the study’s focus on implementing a rehabilitation programme in the context of palliative care is supported by a growing body of evidence highlighting the benefits of incorporating rehabilitation interventions for patients with cancer. Rehabilitation programmes tailored to the individual needs of patients can help to improve physical functioning, reduce symptoms and enhance overall well-being [63, 64]. By addressing the specific functional challenges and impairments faced by cancer patients, rehabilitation programmes can make a significant contribution to improving their quality of life and overall sense of independence [65, 66].

The considerable decrease in anxiety and depression levels detected in patients following their participation in the palliative care rehabilitation programme corresponds with the literature. Patients diagnosed with cancer frequently encounter increased levels of anxiety and depression due to the unsettling character of their diagnosis and treatment. Palliative care provides significant assistance in managing patients’ psychological and emotional needs, supporting their general wellbeing [52, 61, 67]. The observed decrease in anxiety and depression levels confirms the success of the programme in addressing patients’ mental health issues and aligns with the comprehensive approach to palliative care. The decrease in anxiety and depression levels that patients experienced after engaging in the programme is congruent with prior research emphasizing the psychological advantages of palliative interventions [62–65].

Besides, palliative care patients displayed a noteworthy reduction in their anxiety and depression levels, in line with literature. Haun et al.‘s [68] review highlights the effectiveness of palliative care interventions in addressing psychological distress among cancer patients and thereby improving their emotional well-being. This evidence emphasizes the crucial role of palliative care programs in bolstering the quality of life and mental health of cancer patients and underlines the need for these services to be integrated into standard cancer care protocols.

The study’s results indicating a significant and meaningful improvement in the overall quality of life of primary caregivers of cancer patients following the implementation of a rehabilitation palliative care program are consistent with growing evidence highlighting the importance of supporting caregivers in the context of palliative care. Caring for a loved one with cancer can be physically and emotionally demanding, often leading to caregiver distress, burnout, and a reduced quality of life. Palliative care programs recognize the needs of caregivers and provide them with various forms of support, such as counseling, respite care, and education, aimed at reducing caregiver burden and enhancing their quality of life [69, 70].

In addition, the results align with research showing that caregiver support programs can have a positive impact on reducing depression and anxiety levels among caregivers. Palliative care interventions that address the psychological needs of caregivers through counseling and emotional support can lead to improved mental health and overall quality of life [35]. Reducing caregiver distress can also enhance the care they provide to the patient, creating a positive feedback loop that benefits both parties. The integration of rehabilitation into palliative care can further enhance the well-being of caregivers. By offering respite care and teaching caregivers how to assist with physical care and mobility, rehabilitation can reduce the physical and emotional burden of caregiving. This, in turn, contributes to an improved quality of life for caregivers [25].

The results indicating a substantial reduction in the total scores of the Caregiver Burden Inventory and Beck Anxiety Inventory scales following the implementation of a rehabilitation palliative care program are supported by a growing body of research emphasizing the positive effects of palliative care interventions on caregiver well-being and mental health. Caregivers of patients with terminal illnesses often experience significant burden and psychological distress, including anxiety and depression, which can adversely affect their own quality of life [18, 25].

The reduction in total scores of the Caregiver Burden Inventory can be attributed to the comprehensive support provided by palliative care programs, including respite care, counselling, and education. By addressing the practical and emotional challenges faced by caregivers, these programs help alleviate the burden associated with caregiving responsibilities and improve the overall well-being of caregivers [67, 68]. Moreover, the reduction in total scores of the Beck Anxiety Inventory highlights the positive impact of the palliative care program in addressing the psychological distress experienced by caregivers. By providing psychological support and coping strategies, such programs can effectively reduce anxiety levels and improve the mental health of caregivers [10, 15].

Furthermore, the integration of rehabilitation within the palliative care program likely contributes to the observed reduction in caregiver burden and anxiety. Rehabilitation interventions, such as physical therapy and occupational therapy, can alleviate the physical strain associated with caregiving, thereby reducing the overall burden on caregivers [11, 18]. Additionally, the positive effects of exercise and physical activity on mental health are well-documented, further contributing to the reduction in anxiety levels among caregivers [30, 45].

The quasi-experimental investigation examining how a rehabilitation and palliative care programme affects the quality of life of cancer patients and their informal caregivers has certain constraints. Initially, the lack of randomly assigning participants to intervention and control groups may introduce selection bias, as they may possess inherent differences influencing the outcomes. Moreover, the study’s generalizability may be restricted by the small sample size in quasi-experimental designs, and the focus on short-term effects may not fully capture the program’s long-term effects. Self-report measures’ data collection can be influenced by recall bias, and the absence of control over external factors or events may result in confounding results. Additionally, it is possible that the study did not sufficiently account for the potential of self-selection bias among participants, and the utilization of self-report measures may have led to the introduction of social desirability bias. These limitations indicate a requirement for more comprehensive research designs and larger, more varied samples for improved comprehension of the program’s effects on patients and caregivers.

### Recommendations:

1. **Early Integration of Palliative Care:** Based on the study's findings, integrating palliative care early in the cancer care process can significantly improve the quality of life (QOL) for patients. The increase in QOL post-intervention indicates the importance of early and comprehensive palliative care.
2. **Multidisciplinary Approach**: The improvements in both patient and caregiver outcomes suggest that a multidisciplinary team is crucial. Implement a team that includes physicians, nurses, social workers, psychologists, and rehabilitation specialists to address the physical, emotional, and social needs effectively.
3. **Caregiver Education**: Given the significant reduction in caregiver burden and the decrease in anxiety levels, it is essential to develop educational programs for caregivers. These programs should focus on caregiving skills and emotional resilience to enhance their capacity to support patients.
4. **Psychosocial Support**: The study shows a substantial decrease in patients' anxiety and depression, highlighting the need for integrated psychosocial support services. Providing counselling and emotional support is crucial for both patients and caregivers to manage their mental health.
5. **Respite Care**: The reduction in caregiver burden emphasizes the need for respite care options. Offering temporary relief to caregivers can help them manage stress and avoid burnout, ultimately improving their ability to care for patients.
6. **Regular Assessment**: Implement routine assessments of patients and caregivers to tailor interventions effectively. Regular monitoring of QOL and emotional well-being can help adjust care plans to meet evolving needs, as evidenced by the study's positive outcomes.
7. **Physical Rehabilitation**: The study implies that comprehensive rehabilitation, which may include physical rehabilitation, contributed to the improved QOL for patients. Incorporating physical rehabilitation into palliative care can enhance patients' physical and emotional well-being.
8. **Community Resources**: Connecting patients and caregivers with community resources is essential. The significant improvements in caregivers' QOL and reduced burden indicate the importance of external support networks. Facilitating access to community resources can provide additional emotional and practical support.
9. **Continuity of Care**: Ensuring seamless coordination between hospital-based and home-based care is vital. Consistent support throughout the care journey can maintain the improvements in QOL and reduce caregiver stress, as observed in the study.
10. **Ongoing Research**: Continued research is necessary to refine and improve palliative care and rehabilitation programs. The study's positive results underscore the need for ongoing investigation to optimize these interventions and expand their benefits.

## Conclusion

In conclusion, the incorporation of a program for rehabilitation and palliative care has evidenced an extensive and diverse effect on the standard of living of individuals diagnosed with cancer and their non-professional caregivers. The programme’s wide-ranging approach, which covers physical, emotional, and psychosocial aspects of care, has notably upgraded the general welfare of patients through the enrichment of symptom control, pain management, and emotional sustenance. Moreover, the initiative has successfully lessened the burden and anxiety experienced by family caregivers by providing them with the necessary resources, education and respite care to ease the challenges associated with caregiving. Recognising and addressing the distinct requirements of both patients and caregivers within the palliative care framework, this integrated approach has not only enhanced the quality of life for those confronting terminal cancer but has also cultivated a supportive and compassionate environment that facilitates superior outcomes and a sense of solace for all parties involved.

## Data Availability

The datasets used and/or analyzed during the current study are available from the corresponding author on request.

## Abbreviations

BAI: Beck Anxiety Inventory
CBI: Caregiver Burden Inventor
EORTC QLQ-C30: European Organization for the Research and Treatment of Cancer Quality of Life Questionnaire 30
GLOBOCAN: Global Cancer Observatory
HCV: Hepatitis C virus
HADS: Hospital Anxiety and Depression Scale
NICE: National Institute for Health and Care Excellence
QOL: Quality Of Life
SF-36: Short Form Health Survey
WHO: World Health Organization
